# Simultaneous co-cultivation of the thermoacidophilic methanotroph, *Methylacidiphilum* sp. RTK17.1, and the microalga, *Galdieria* sp. RTK37.1, for single cell protein production

**DOI:** 10.1016/j.engmic.2025.100229

**Published:** 2025-08-06

**Authors:** Carlos Cartin-Caballero, Christophe Collet, Daniel Gapes, Peter A. Gostomski, Matthew B. Stott, Carlo R. Carere

**Affiliations:** a Te Tari Pūhanga Tukanga Matū |Department of Chemical and Process Engineering, Te Whare Wānanga o Waitaha |University of Canterbury, Christchurch 8140, New Zealand; b Scion, Te Papa Tipu Innovation Park, Rotorua, New Zealand; c Cetogenix, c/o Scion, Private Bag 3020, Rotorua 3046, New Zealand; d Te Kura Pūtaiao Koiora-School of Biological Sciences | Te Whare Wānanga o Waitaha | University of Canterbury, Christchurch 8140, New Zealand; e CIIBio, Universidad Nacional, Costa Rica

**Keywords:** Microalgae, Methanotroph, Coculture, Bioprocess, Extremophile, Single cell protein

## Abstract

The verrucomicrobial methanotroph, *Methylacidiphilum* sp. RTK17.1, and the microalgae, *Galdieria* sp. RTK37.1 are both thermoacidophilic microorganisms isolated from geothermally heated soils at Rotokawa, Aotearoa-New Zealand. In this work, we used cocultures of *Methylacidiphilum* sp. RTK17.1 and *Galdieria* sp. RTK37.1 in batch and continuous systems (45 °C, pH 2.5) to assess their biomass productivity and performance; with the goal of removing methane and carbon dioxide from simulated waste gas streams and assessing the resultant biomass for its potential use as single cell protein. Coculture performance was compared to corresponding axenic cultures and the nutritional suitability of resultant biomass was assessed as a single cell protein feedstock. Stable coculture was achieved in both batch and chemostat systems. In batch experiments, *Galdieria* sp. RTK37.1 significantly enhanced growth (29 %) and methane oxidation (300 %) rates of *Methylacidiphilum* sp. RTK17.1, and complete methane removal was achieved without formation of an explosive gas mixture. In steady state chemostat coculture experiments, *Galdieria* sp. RTK37.1 decreased net volumetric oxygen consumption by 46 %, but its oxygenic activity was unable to supply *Methylacidiphilum* sp. RTK17.1 with the O_2_ required for complete CH_4_ removal. Nevertheless, *Methylacidiphilum* sp. RTK17.1 benefited from the presence of *Galdieria* sp. RTK37.1 in a low O_2_ environment; with O_2_ algae-methanotroph cross-feeding playing a fundamental role on their interactions. *Methylacidiphilum* sp. RTK17.1, *Galdieria* sp. RTK37.1, and their coculture each displayed similar nutritional profiles, with protein quality comparable to soybean meal and fishmeal feeds used for animals. The biomass needed to meet the daily indispensable amino acid requirements of a 62 kg adult human was 568 g for *Methylacidiphilum* sp. RTK17.1, 804 g *Galdieria* sp. RTK37.1, and 754 g for the coculture, with histidine being the limiting amino acid. These thermoacidophilic cocultures, which have not previously been investigated, offer great potential to convert low (or negative) value industrial gas streams into valuable products (e.g. supplementary biofeedstocks).

## Graphical abstract

**Figure:**
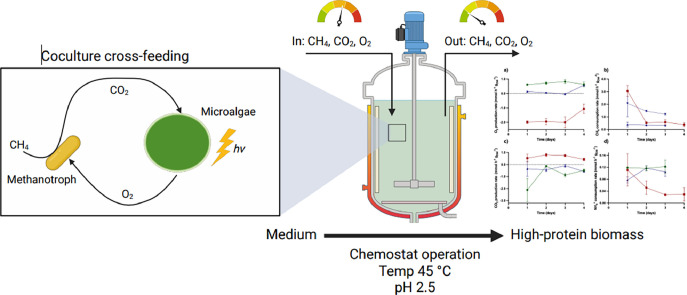

## 1 Introduction

Methane (CH_4_) is the most abundant organic gas in the atmosphere [1] and, behind carbon dioxide (CO_2_), is the second most impactful anthropogenic greenhouse gas [2]. While CO_2_ abatement is the focus of most climate change control strategies, CH_4_ emission reduction is similarly vital to achieve rapid and significant impacts on atmospheric warming, given methane's greater heat-trapping efficiency and shorter atmospheric lifetime compared to CO_2_ [3]. Aerobic methane oxidizing bacteria (methanotrophs) consume CH_4_ as their sole carbon and energy source [1] and are the primary biological sink for this climate active gas [1,4]. Industrial bioprocesses using methanotrophs could potentially use the CH_4_ in waste flue gas emissions for growth, with the produced biomass representing a source of single cell protein (SCP) for use as animal feed [5,6]. Biomass from the methanotroph *Methylococcus capsulatus* is commercially available as SCP [7], and natural gas as a microbial feedstock is used to produce feed for farmed salmon [3]. *M. capsulatus* SCP has been shown to reduce potentially pathogenic gut bacteria and boost immunity in fish [8], while also potentially contributing to antibiotic resistance mitigation by replacing protein sources tied to antibiotic use [9,10]. Although methanotrophic cultivation offers several advantages for SCP production, methane oxidation by aerobic methanotrophs requires O_2_ supplementation, which presents safety concerns for bioprocess development as CH_4_-air mixtures are explosive between 5 % to 15 % CH_4_ (v/v) [11]. A potential solution to this problem is the coculture of methanotrophs with oxygenic photoautotrophic microalgae. It is anticipated that simultaneous O_2_ production and consumption, *in situ,* can mitigate the development of explosive gas (CH_4_-Air) mixtures.

Microbial species interactions in cocultures can be stimulating, inhibitory, or neutral [12]. Stimulating interactions are usually established via nutrient exchange [13] and include mutualistic and commensal relationships. Inhibitory interactions usually arise from resource and/or spatial competition, and can produce secondary metabolites that give advantage one of the species in detriment to the other [14]. Methanotrophic bacteria can benefit from co-cultivation with other microorganisms [15]. For example, co-cultivation with the heterotroph, *Sphingopyxis* sp. NM1, stimulates the growth of the methanotroph, *Methylocystis* sp. M6, in a concentration dependant manner [15]. A similar effect was found in cocultures of *Hyphomicrobium* sp. NM3 and *Methylocystis* sp. M6 [16]. However, while the oxidation rate and biomass of the methanotroph increased, *Hyphomicrobium* populations tended to decrease with time [16]. Biomass production of both *Rhizobium* sp. Rb122 and the methanotroph *Methylovulum miyakonse* HT12 increased during co-cultivation; with cobalamin production by *Rhizobium* identified as a stimulating factor [17]. In another study, *Methylococcus capsulatus* benefited from coculturing with *Ralstonia* sp., *Brevibacillus agri* and *Aneurinibacillus* sp., as these species removed accumulated acetate and other inhibitory organic material [18].

Cocultures of photoautotrophs with heterotrophic bacteria often involve mutualistic nutrient exchanges. Microalgae commonly provide O_2_ and organic compounds via photosynthesis while bacteria, in turn, produce CO_2_ and other inorganic metabolites via respiration [13]. Co-cultivation of unspecified methane-oxidizing communities with microalgae obtained from open ponds exhibit faster CO_2_ fixation rates and require 40–55 % less oxygen supplementation [19]. Cocultures of *M. capsulatus* and the microalgae *Chlorella sorokiniana* can produce single cell protein (SCP) using potato-processing wastewater [20]. Furthermore, in illuminated chemostat coculture using synthetic natural gas as a growth substrate, the cyanobacterium *Synechococcus* PCC 7701 produced enough O_2_ to support the growth of *Methylomicrobium alcaliphilum* 20z despite negligible dissolved O_2_ [21].

The use of industrial flue gas emissions as a substrate for microbial co-culture bioprocesses is challenged by their hot temperature, the potential toxicity of CO_2_, NO_x_ and SO_x_ at industrially relevant concentrations [[22], [23], [24]] and the tendency for these gases to acidify media [[25], [26], [27]]. While few algae are viable under these harsh conditions, the thermoacidophilic red algae *Galdieria* spp. are often found in similar hostile environments; including volcanic hot sulphur springs, solfatara soils and geothermal environments [[28], [29], [30]]. Likewise, thermoacidophilic verrucomicrobial methanotrophs (e.g. *Methylacidiphilum* spp. and *Methylacidimicrobium* spp.) are often co-located in these extreme environments [[30], [31], [32]].

In this study, we investigate the coculturing of thermoacidophilic methanotroph and algae strains and their potential use to convert industrial flue gas emissions into single cell protein for animal feed. The verrucomicrobial methanotroph, *Methylacidiphilum* sp. RTK17.1, and the microalga, *Galdieria* sp. RTK37.1 were both previously isolated from soils within the Rotokawa geothermal region in Aotearoa-New Zealand [33,34] and display broadly similar pH and temperature optima. *Methylacidiphilum* sp. RTK17.1 oxidizes CH_4_, reduces CO_2_ via the Calvin-Benson-Bassham cycle, fixes N_2_ under nitrogen-limiting conditions, accumulates glycogen, and rapidly consumes H_2_ under microaerophilic conditions [35,36]. *Galdieria* sp. RTK37.1 is phenotypically very similar to the characterised *Galdieria sulphuraria* and is capable of photoautotrophic (via the Calvin-Benson-Bassham), mixotrophic and heterotrophic growth [24,29,33]. No prior studies on the co-cultivation of these thermoacidophilic taxa exist and little is known about their possible mutualistic interactions, or their suitability for feed applications. Therefore the aim of this study was to assess if *Methylacidiphilum* sp. RTK17.1 and *Galdieria* sp. RTK37.1 could be grown in coculture, to compare the differences between their axenic and coculture growth, and to test their suitability as single cell protein feedstocks.

## 2 Materials and methods

### 2.1 Growth medium and culture maintenance

A modified V4 medium [31] was used for all cultivation experiments. Briefly, the medium contained per litre: 0.4 g NH_4_Cl, 0.05 g KH_2_PO_4_, 0.02 g MgSO_4_·7H_2_O, 0.01 g CaCl_2_·2H_2_O, 3 mL FeEDTA solution, 3 mL trace elements solution, 1 mL trace metals solution, 0.2 µM Ce_2_(SO_4_)_3_, and 0.2 µM La_2_(SO_4_)_3_ solution and was adjusted to pH 2.5 with H_2_SO_4_. Unless stated otherwise, all chemicals and reagents were purchased from Sigma-Aldrich (Germany) and all gases were sourced from BOC (Australia). Previously, both *Methylacidiphilum* sp. RTK17.1 and *Galdieria* sp. RTK37.1 were isolated from geothermally heated soils at Rotokawa, Aotearoa-New Zealand [33,34]. As previously described, *Methylacidiphilum* sp. RTK17.1 was routinely maintained in chemostat culture using a 1 L bioreactor (600 mL working volume, BioFlo 110; New Brunswick Scientific, United States) with a 0.0069 h^−1^ dilution rate at 50 °C, agitation at 800 rpm, and pH ∼ 2.5 (but not controlled) [34]. A custom gas mixture (69 % CO_2_, 1.0 % CH_4_, 3.1 % O_2_, balance N_2_, all v/v) was supplied at 20 mL min^−1^. Under these conditions, typical steady-state OD_600_ values were 1.2 A.U. and outlet gas concentrations were 72 % CO_2_, 0.13 % CH_4_, 1.5 % O_2_, with the remaining attributed to N_2_. For all experiments, *Methylacidiphilum* sp. RTK17.1 was aseptically harvested directly from the reactor and diluted with sterile V4 medium to the desired starting concentration.

For *Galdieria* sp. RTK37.1, 300 mL stocks were routinely grown in batch within 1 L Duran^Ⓡ^ Pressure Plus Bottles (Sigma-Aldrich, Germany) equipped with bromobutyl rubber stoppers. At the beginning of each cycle, the microalgae would be diluted to OD_600_ 1.0 with sterile V4 medium, the bottles were then subjected to a vacuum for 3 min, and re-pressurized to 5 psia with an 80 % v/v CO_2_ and 20 % v/v N_2_ gas mixture. The bottles were incubated horizontally in a Lab Companion shaking incubator (Cole-Parmer, Illinois, United States) at 110 rpm and 45 °C. Light was provided via three 50 W halogen lightbulbs and adjusted to 60 µmol_photons_ *m*^−2^ s^−1^ measured at bottle’s level with a LI-250A Light Meter (LI-COR, Nebraska, United States). Growth was stopped at OD_600_ 6.0 and the cycle started anew. For all experiments, *Galdieria* sp. RTK37.1 was aseptically harvested at a OD_600_ 5.0 A.U. and diluted with sterile V4 medium to the desired starting concentration.

### 2.2 Coculture batch experiments

*Methylacidiphilum* sp. RTK17.1 and *Galdieria* sp. RTK37.1 were grown together in batch to assess coculture viability and to investigate biomass productivity relative to axenic controls. For these experiments, 250 mL of sterile V4 medium was added to 1 L Duran^Ⓡ^ Pressure Plus Bottles sealed with bromobutyl rubber stoppers. For the cocultures, *Galdieria* sp. RTK37.1 was inoculated to a starting OD_600_ of 0.9 and *Methylacidiphilum* sp. RTK17.1 to OD_600_ 0.1. Contemporaneously, axenic microalgae controls were inoculated to a starting OD_600_ of 1.0 and axenic methanotroph controls to OD_600_ 0.1. Coculture headspaces were prepared by subjecting the bottle to vacuum for 3 min and then re-pressurizing to 5 psia with an 80 % CO_2_ and 20 % N_2_ gas mixture (v/v) three times. After this, headspace gas was equilibrated to atmospheric pressure by expanding against a syringe, and then 120 mL of 100 % CH_4_ (v/v) was injected. The headspace gas was then released to 10 kPa. The axenic *Galdieria* sp. RTK37.1 headspace was prepared in a similar fashion, but without CH_4_ addition. The headspace air was not replaced for the *Methylacidiphilum* sp. RTK17.1 controls, but 120 mL of CH_4_ and 60 mL of CO_2_ were injected, and then the pressure released to 10 kPa (Table S1). All batch cocultures and controls were run in duplicate. All bottles were incubated horizontally at 110 rpm and 45 °C with light intensity adjusted to 60 µmol_photons_ *m*^−2^ s^−1^ as described above.

To assess cell growth and gas production/consumption rates respectively, daily 2 mL liquid and 10 mL headspace gas samples were collected. Headspace pressure was measured with an Almelmo 5470 data logger (Ahlborn, Germany) equipped with a pressure sensor (Gems Sensors & Controls, England) before and after all headspace sampling to account for gas loss. To minimise glycogen accumulation, experiments were stopped before ammonium depletion, or the onset of stationary phase [36]. To harvest biomass for nutritional analysis, the replicates were combined and centrifuged at 12,000 rpm on a 5810R Benchtop Centrifuge (Eppendorf, Hamburg) for 15 min. Supernatants were discarded, and each biomass pellet was stored at −20 °C until needed. To assess the effect on glycogen accumulation on the microalgae’s protein concentration, three additional bottles of *Galdieria* sp. RTK37.1 were grown, as described above, but allowed to reach stationary phase (OD_600_ = 9.25 ± 0.3). Insufficient axenic *Methylacidiphilum* sp. RTK17.1 biomass was obtained from these batch experiments to allow for its nutritional characterization.

### 2.3 Coculture chemostat experiments

To evaluate continuous biomass productivity of cocultures, and to generate sufficient biomass for amino acid characterization, three chemostats were operated in parallel for each axenic culture and coculture. For each, a 1 L bioreactor (900 mL working volume, BioFlo 110, New Brunswick Scientific, Edison, NJ, USA) was used [34], cultures were incubated at 45 °C and sterile V4 medium at pH 2.5 provided. For the axenic *Methylacidiphilum* sp. RTK17.1 and the coculture bioreactors, 150 mL day^−1^ V4 medium was supplied (*D* = 0.167 day^−1^) with agitation maintained at 250 rpm. Due to technical constraints, axenic *Galdieria* sp. RTK37.1 chemostat cultivation was performed at 400 rpm and *D* = 0.278 day^−1^. Dissolved O_2_ was monitored using an InPro 6810 Polarographic Oxygen Sensor (Mettler-Toledo, Columbus, OH, USA) and pH was monitored with a Polilyte Plus pH Meter (Hamilton Company, United States). Custom gas mixtures were prepared, as required, in compressed gas cylinders and supplied at 16 mL min^−1^ using mass flow controllers (El-flow, Bronkhorst, Netherlands). Feed gas was filter sterilised before entering the reactors using in-line 0.22 µm hydrophobic filters (MicroScience, Australia). Feed gas composition for *Methylacidiphilum* sp. RTK17.1 and the coculture were approximately 2.1 % O_2_, 1.1 % CH_4_, 74 % CO_2_, balance N_2_, (all v/v). For *Galdieria* sp. RTK37.1, feed gas composition was 3.0 % O_2_, 64 % CO_2_, balance N_2_. Light was supplied to the axenic microalgae and coculture bioreactors via warm white LED strips attached to the walls of the reactor at an intensity adjusted to 100 µmol_photons_ *m*^−2^ s^−1^ measured at the centre of the empty reactor. To initiate axenic culture experiments, reactors were inoculated to OD_600_ 0.1 with the corresponding microorganism and grown in batch mode until an OD_600_ of 1.0 A.U. was reached; after which the inlet and outlet peristaltic pumps were activated (Cole-Parmer, Illinois, United States). For the coculture experiment, the reactor was inoculated to 0.1 OD_600_ of *Galdieria* sp. RTK37.1, grown in batch until OD_600_ = 1.0 A.U. and then the pumps were started. After achieving steady state, < 1 mL *Methylacidiphilum* sp. RTK17.1 (OD_600_ = 1.20 A.U.) was added to the reactor. For all reactors, daily samples (2 mL liquid and 40 mL inlet and outlet gas samples) were aseptically harvested for biomass (OD_600_) and gas measurements. Systems were considered at ‘steady state’ when there was < 5 % OD_600_ change over a five-day period. For nutritional analysis the steady state outflow broth was collected daily and stored at 4 °C. After 1 L of broth had accumulated, it was centrifuged at 12,000 rpm in a 5810R Benchtop Centrifuge (Eppendorf, Hamburg) for 15 min. Supernatants were then discarded, and the resulting biomass pellets were stored at −20 °C until needed.

### 2.4 Methylacidiphilum sp. RTK17.1 cultivation in 10 L bioreactor

To produce sufficient biomass (> 5 g_DW_) for complete amino acid characterization (including tryptophan), *Methylacidiphilum* sp. RTK37.1 was cultivated in a batch 10 L bioreactor. For this cultivation, 1 L of the effluent from the axenic *Methylacidiphilum* sp. RTK17.1 chemostat was used to inoculate a 10 L Biostat D-DCU STR reactor (Sartorius, Germany). Prior to inoculation, the reactor containing 10 L V4 medium was sterilized by autoclaving for 20 min at 121°C. A custom gas mixture (12.3% CO_2_, 2.5% CH_4_, 7.9% O_2_, balance N_2_, all v/v) was supplied continuously at 800 mL min^−1^. The inlet gas mixture was sterilized via a Sartufluor GA Cartridge inline single-layer PTFE membrane filter (0.2 µm pore size, Sartorius, Germany). Throughout cultivation (220 h total), the temperature was maintained at 50°C, agitation at 500 rpm, and pressure at 100 mbar_g_. To ensure cells never experienced nitrogen limitation, 4 g of NH_4_Cl dissolved into 50 mL deionized water was added, via filter sterilisation (0.2 µm pore size), at OD_600_ = 3.0 (120 h) and OD_600_ = 6.0 (198 h). Following 220 h incubation, 9.1 L was harvested (OD_600_ = 9.2 A.U.) which was then centrifuged for 10 min at 8500 rpm on a Sorvall RC6 Plus centrifuge (Thermo Scientific, United States) and subsequently stored at -80 °C for 24 h to prepare for freeze drying. Finally, the cell mass was freeze dried for 72 h using an Edwards SuperModulyo Freeze Dryer (Edwards, Sweden),and then stored at -20 °C until required for nutritional analysis.

### 2.5 *Galdieria* sp. RTK37.1 cultivation in 80 L tubular photobioreactor

*Galdieria* sp. RTK37.1 was cultivated in an 80 L tubular photobioreactor to produce sufficient biomass (> 5 g_DW_) for complete amino acid characterization (including tryptophan) in batch culture. Briefly, the inoculation train for microalgae first involved cultivation in 250 mL baffled shake flasks (100 mL sterile V4 medium, 45 °C, pH 2.5) inside a MaxQ 6000 shaking incubator (Thermo Fisher Scientific, USA) modified with LED lights (warm white, 103 umol_photons_ *m*^−2^ s^−1^) and a gas manifold system able to continuously supply air supplemented with 3 % CO_2_ (25 mL min^−1^). Next, when *Galdieria* sp. RTK17.1 shake flasks reached OD_600_ 6.4, it was used to inoculate a 1.5 L concentric tube airlift bioreactor containing 1350 mL sterile V4 medium. Airlift bioreactors were operated as described previously [33]. When the OD_600_ reached 6.6, the entire contents of the airlift bioreactor were used to inoculate an 80 L tubular photobioreactor.

The custom 80-L tubular photobioreactor consisted of a bubble column and a solar collector [37]. The bubble column had an internal diameter of 140 mm and a height of 1800 mm and was equipped with a ring-type sparger at the centre of the base of the column, a stainless-steel heat exchanger, and pH, D.O., and temperature probes. The final working volume of the reactor was approximately 76 L with 23 L in the bubble column and 53 L in the solar receiver. The solar receiver was constructed of 12 borosilicate glass tubes (Schott, Germany) of 1500 mm length and 50.4 mm internal diameter. The culture was circulated using a variable-speed centrifugal pump. Lighting was provided by RGBWW LED strips attached to the inside of 100 mm PVC pipe which was split lengthwise to be able to clam shell around each of the solar receiver tubes. Each lighting tube contained a total length of 19.5 m of LED strips. Air and CO_2_ flow rates were controlled with mass flow controllers (Alicat Scientific, USA). Air was supplied from an air compressor and food grade CO_2_ was supplied from cylinders (BOC, New Zealand). All gases were filtered with an inline 0.2 μm PTFE filter (WhatmanTM Polycap 75 TF, GE Healthcare, USA) prior to entering the bubble column to maintain sterility.

V4 growth medium, water and concentrated chemical stock solutions were transferred into the photobioreactor via a peristaltic pump through an inline 0.2 μm filter. The medium was then circulated though the photobioreactor at 0.6 m *s*^−1^ liquid velocity. Compressed air and CO_2_ were supplied at a total flowrate of 7.6 L min^−1^, and a final CO_2_ concentration of 3 % (v/v). Light intensity was set to 100 µmol_photons_ *m*^−2^ s^−1^, and then increased to 300 µmol_photons_ *m*^−2^ s^−1^ after 5 days of cultivation. On day 19, prior to ammonium depletion (OD_600_ = 3.6), 24 L was harvested. To analyse the macromolecular and amino acid content of nitrogen-depleted *Galdieria* sp. RTK37.1, the reactor was supplemented with sterile reverse osmosis water to re-establish a 76 L working volume. The culture was then allowed to continue for an additional 7 days, before harvesting in its entirety on day 26 (OD_600_ = 4.7). Next, the harvested *Galdieria* sp. RTK37.1 (both nitrogen-excess and nitrogen-limited) were separately vacuum filtered, with the resultant biomass pastes then washed with deionised water before collection and storage at –80 °C, until required for freeze drying via a Freezone 2.5 freeze-dryer (Labconco, USA). Finally, the freeze-dried biomass was stored at −80 °C until needed for nutritional analysis.

### 2.6 Analytical procedures

Optical density at 600 nm was routinely measured using an Ultrospec 10 cell density meter (Amersham Bioscience, United Kingdom). Biomass concentrations in the batch bottle experiments were calculated using conversion factors previously obtained in axenic cultures (1 OD_600_ = 0.308 g_DW_ *L*^−1^ *Galdieria* sp. RTK37.1, 1 OD_600_ = 0.435 g_DW_ *L*^−1^ *Methylacidiphilum* sp. RTK17.1)*.* For coculture experiments, relative amounts of methanotroph and microalgae were estimated using the oxygen uptake/production rate of the axenic cultures and the oxygen production of the coculture. For the chemostat bioreactors, biomass concentrations were measured gravimetrically. For this, triplicate 50 mL aliquots were harvested and centrifuged at 12,000 rpm in a 5810R Benchtop Centrifuge (Eppendorf, Hamburg) for 15 min. After discarding supernatants, the pellets were resuspended in deionized water and centrifuged again for 15 min. Supernatants were then discarded and the pellets transferred into pre-weighed aluminium dishes before drying overnight at 95 °C. The aliquot biomass weight was calculated by difference.

Gas samples were analysed for CO_2_, CH_4_, N_2_, and O_2_ determinations using a 490 Micro GC equipped with a thermal conductivity detector (Agilent Technologies, USA), a Molecular Sieve 5A with a heated injector (50 °C, back-flush at 5.10 s, column at 90 °C, 150 kPa), a PoraPak Q column with a heated injector (50 °C, no back-flush, column at 70 °C, 50 kPa) and a 5CB column with a heated injector (50 °C, no back-flush, column at 80 °C, 150 kPa).

Ammonium concentrations were monitored using the orthophthaldialdehyde (OPA) method [38] on liquid samples pre-filtered through 0.2 µm nylon syringe filters. Briefly, buffered phthalaldehyde-mercaptoethanol reagent was prepared by mixing 4.5 mL of phtaladehyde solution (10 mg mL^−1^ in 100 % ethanol), 4.5 mL of mercaptoethanol solution (5 µL mL^−1^ in 100 % ethanol), and 81 mL of 0.2 sodium phosphate buffer (pH = 7.4). A 50 µL aliquot of sample was added to 1.45 mL of the buffered OPA reagent. The mixture was reacted for 45 min at room temperature. The fluorescence was then measured at a 410 nm excitation and 470 nm emission in a spectrophotometer (Varioskan Lux, Thermo Scientific, United States). The ammonia fluorescence was corrected for the fluorescence of a deionized water blank. A calibration curve with ammonium chloride standards was run with every set of samples.

Nutritional characterization of biomass was performed by the Massey University Nutrition Laboratory according to the official methods of analysis of the Association of Official Analytical Communities (AOAC, 2005) international. Ash content was determined by the furnace method [39] (AOAC method 942.05), total crude protein was determined via the Dumas method [40] (AOAC method 968.06), fat content was determined by the Mojoinnier method [41] (AOAC method 922.06), and carbohydrates were determined by difference. Amino acid profile determination of acid-stable residues was performed via reverse-phase high performance liquid chromatography (HPLC) separation using AccQ derivatization of biomass (60–140 mg) samples following oxidization with performic acid, and hydrolysis with hydrochloric acid as described in AOAC method 994.12 [42] (AOAC, 2005). Cysteine and methionine content was determined by performic acid oxidation [43] (AOAC method 985.28).

All statistical analyses were performed using Prism Graphpad 9.4.1. Indispensable Amino Acid contents and specific growth rates were compared using unpaired two-tailed *t*-tests (α = 0.05). For all other analysis, unless stated otherwise, Two-factor ANOVA (α = 0.05) tests were used, with Sidak method for multiple mean comparisons between columns (simple effects within rows) or rows (simple effects within columns) implemented as necessary. Growth rates were calculated by fitting biomass data to an exponential growth model in Prism Graphpad 9.4.1.

## 3 Results and discussion

### 3.1 O_2_-dependent cross-feeding enhances CH_4_ removal in batch coculture experiments

**Fig. 1:**
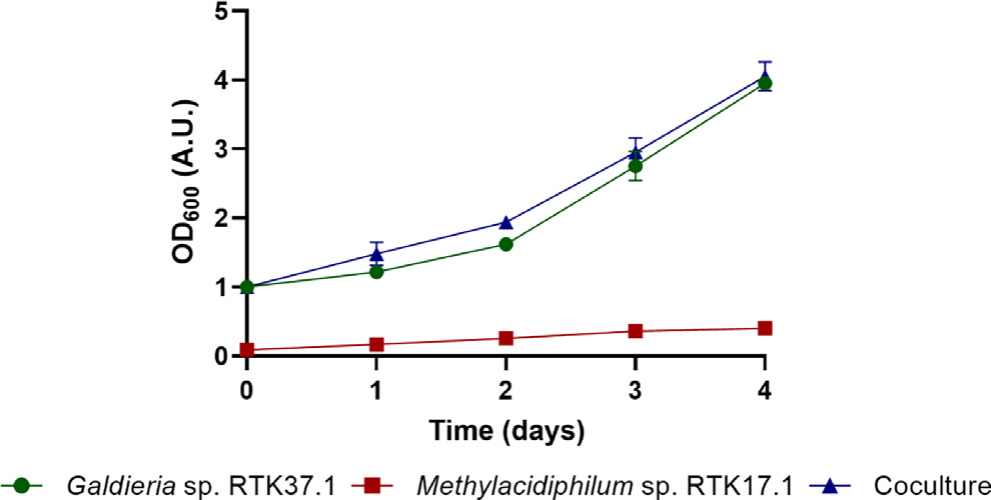
Batch coculture growth of *Methylacidiphilum* sp. RTK17.1 and *Galdieria* sp. RTK37.1 (Coculture) compared to corresponding axenic control experiment. All cultures were grown in 1 L gastight bottles on a shaking incubi tor at 45 °C, pH 2.5, 110 rpm, and 60 μmol *m*^−2^ s^−1^ measured at the top of the bottle wall. The average value of duplicate experiments (*n* = 2) is shown with error bars representing one standard deviation.

**Fig. 2:**
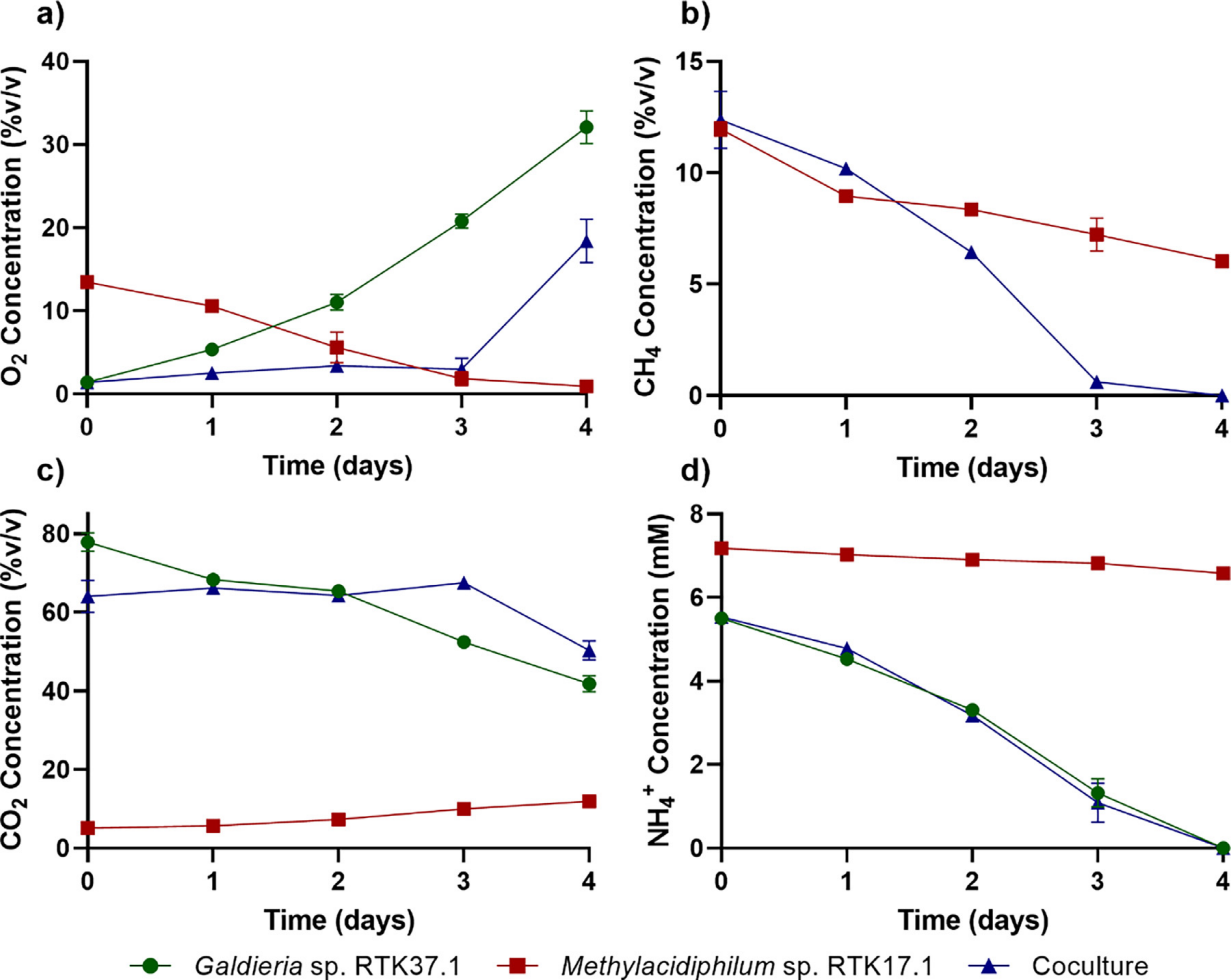
Concentration profile comparison between cocultures of *Methylacidiphilum* sp. RTK17.1 and *Galdieria* sp. RTK37.1 (Coculture) and their corresponding axenic controls. a) O_2_ headspace concentration. b) CH_4_ headspace concentration. c) CO_2_ headspace concentration. d) NH_4_^+^ concentration. All batch cultures were grown in 1 L gastight bottles at 45 °C, pH 2.5, 110 rpm, and 60 μmol *m*^−2^s^−1^ measured at the top of the bottle wall. The average value of duplicate experiments (*n* = 2) is shown with error bars representing one standard deviation. Negative controls displayed no significant gas loss over the cultivation period (not shown).

Initial batch coculture experiments indicated that the oxygenic activity of *Galdieria* sp. RTK37.1 was sufficient to support the aerobic oxidation of CH_4_, by *Methylacidiphilum* sp. RTK17.1, without the need for O_2_ supplementation. As expected, the onset of stationary phase in axenic methanotroph batch cultures corresponded to an O_2_ limitation in the headspace (0.174 ± 0.013 g_DW_ *L*^−1^, < 0.9 % O_2_, Fig. 1, Fig. 2). In contrast, in cocultures continuous O_2_ evolution by the microalgae permitted complete CH_4_ consumption (LDL < 0.01 % CH_4_) within 3 days incubation. Spectrophotometric measurements showed growth of the coculture was comparable to microalgae controls, without any indication of inhibitory bacterial shading at the cell concentrations and bacteria/microalgae ratios used (Fig. 1). This contrasts prior cocultures of *Galdieria sulphuraria* UTEX 2919 with a mixed bacterial community from industrial wastewater, where biomass concentrations decreased, at high biomass concentrations, in coculture due to bacterial shading and O_2_ limitation [44].

The specific growth rate for *Methylacidiphilum* sp. RTK17.1 in coculture (0.022 h^−1^) was significantly faster than in the axenic control (0.017 h^−1^, *p*-value < 0.05). In other non-extremophilic methanotroph-photoautotroph cocultures, photoautotrophs tend to benefit their partner (e.g. faster growth and/or CH_4_ oxidation rates) via O_2_ supplementation from photosynthetic activity [45,46]. Van der Ha et al. [19] reported a 1.6x increase in biomass yield of a methane-oxidizing community from anaerobic sludge tanks when cocultured with a microalgal community dominated by *Scenedesmus* strains. Cocultures of *Methylomicrobium alcaliphilum* 20z with the cyanobacterium *Synechococcus* PCC 7002 resulted in greater biomass concentrations and slightly slower specific growth rates [21]. Similarly, despite much slower growth rates, the final stationary phase biomass concentration of a batch coculture including *Methylococcus capsulatus* and the microalga *Chlorella sorokiniana* was 2.17x greater compared to axenic counterparts [20].

**Fig. 3:**
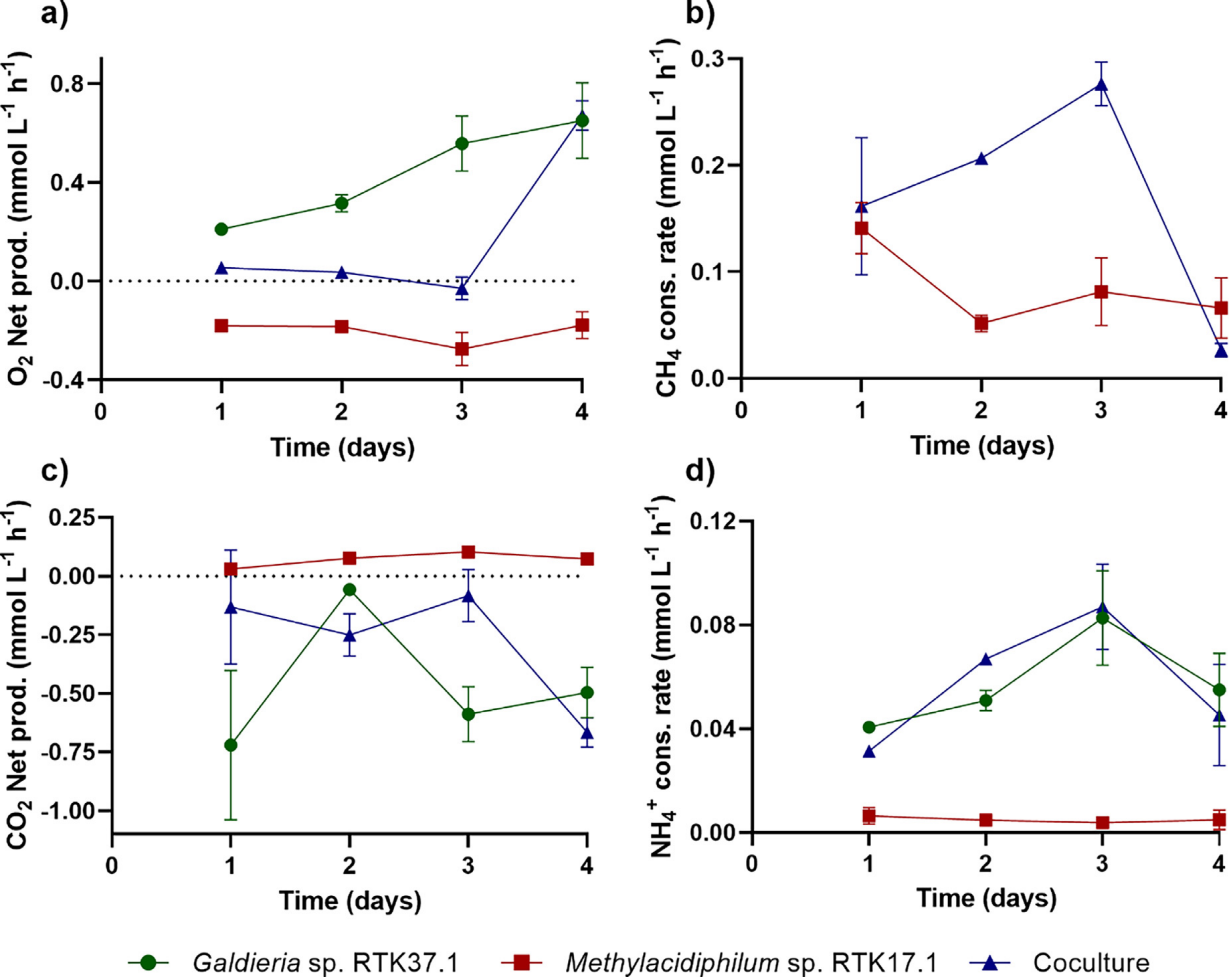
Production and consumption rates comparison between cocultures of *Methylacidiphilum* sp. RTK17.1 and *Galdieria* sp. RTK37.1 (Coculture) and their axenic controls. a) O_2_ net production rates. b) CH_4_ consumption rates. c) CO_2_ net production rates. d) NH_4_^+^ consumption rates. Batch cultures were grown in 1 L gastight bottles at 45 °C, pH 2.5, 110 rpm, and 60 μmol *m*^−2^ s^−1^ warm white light (measured at the top of the bottle wall). The average value of duplicate experiments (*n* = 2) is shown with error bars representing one standard deviation.

Gas concentration profiles indicate O_2_-dependent cross feeding enhanced CH_4_ removal in batch experiments (Fig. 2). In axenic cultures, O_2_ was steadily accumulated by *Galdieria* sp. RTK37.1 and depleted by *Methylacidiphilum* sp. RTK17.1 (Fig. 2a). In contrast, the O_2_ concentration in cocultures remained stable (between 1.4 % to 3.4 % v/v) until CH_4_ depletion on day 3 (Fig. 2b). Methane consumption rates within axenic *Methylacidiphilum* sp. RTK17.1 cultures (Fig. 3b) slowed following the first day, presumably due to O_2_ limitation in the liquid phase. In contrast, coculture CH_4_ oxidation rates increased continuously until CH_4_ was depleted. The cessation of CH_4_ oxidation in the coculture coincided with rapidly increasing O_2_ concentrations (Fig. 2, Fig. 3). In batch, *Methylacidiphilum* sp. RTK17.1 quickly became O_2_-limited during axenic growth, and when active in cocultures, impeded O_2_ gas accumulation, in coculture. As < 2 % (v/v) O_2_ was initially present in the coculture, the stoichiometric O_2_ requirement for CH_4_ oxidation dictates that both *Methylacidiphilum* sp. RTK17.1 and *Galdieria* sp. RTK37.1 were metabolically active throughout the incubation period. Prior studies have also reported CH_4_ oxidation in the absence of supplemental O_2_ addition [21]. Therefore, not only is coculture possible, but the methanotroph benefits from this interaction in low O_2_ environments; without formation of an explosive gas mixture [47].

In this study ammonium (NH_4_^+^) was used as the primary anabolic nitrogen source for both microorganisms. *Methylacidiphilum* spp. fix N_2_ under nitrogen-limiting conditions, when O_2_ concentrations are < 2 % v/v [48]. Furthermore, both *Galdieria* spp. and *Methylacidiphilum* spp. accumulate glycogen, to the detriment of protein yields, under nitrogen starvation conditions [33,36, [48], [49], [50]]. Therefore, as either N_2_-fixation and/or glycogen production would complicate comparisons between cultures, experiments were stopped prior to ammonium depletion. Ammonium consumption rates (Fig. 3d) were steady for *Methylacidiphilum* sp. RTK17.1 cultures and relatively slow when compared to *Galdieria* and coculture experiments, thus the presence of the methanotroph did not significantly impact the coculture’s ammonium uptake (*p*-value > 0.05).

Although elevated CO_2_ concentrations have been found to inhibit growth in cocultures of *M. alcaliphilum* 20z and *Synechococcus* PCC 7002 [21], *Methylacidiphilum* spp. and *Galdieria* spp. are reported to grow without inhibition up to 92.7 % and 100 % CO_2_ respectively [24,48]. In this study, neither the axenic *Galdieria* sp. RTK37.1, nor the coculture showed signs of CO_2_ inhibition. While periods of methanotrophic activity corresponded to no net O_2_ production/consumption in the coculture, a modest net consumption of CO_2_ was observed (Fig. 3c). This is consistent with reports that verrucomicrobial methanotrophs fix CO_2_ via the Calvin Benson cycle [48,51]. Thus, a proportion of the CO_2_ produced during CH_4_ oxidation in coculture was assimilated into both new *Methylacidiphilum* sp. RTK17.1 and *Galdieria* sp. RTK37.1 biomass.

**Fig. 4:**
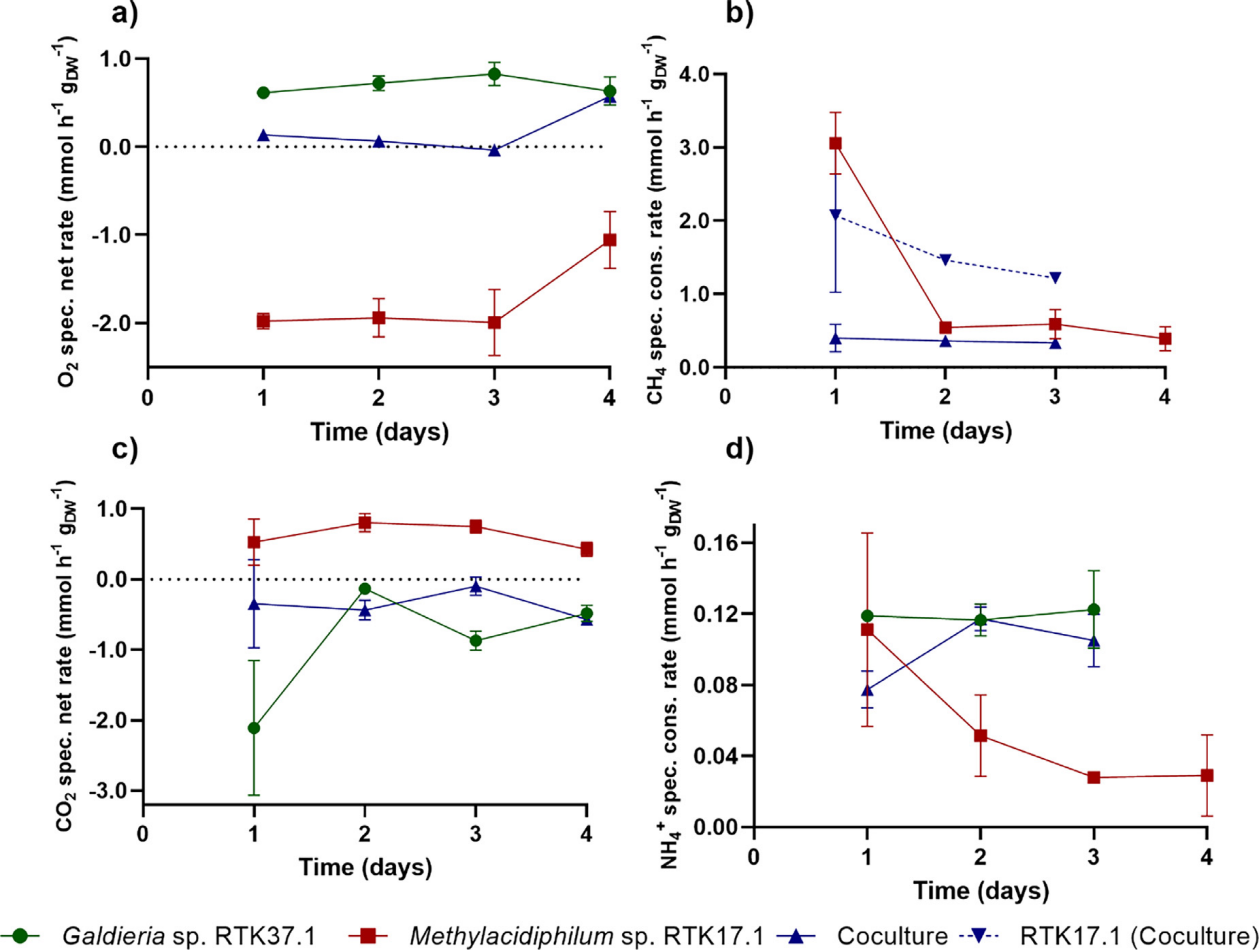
Production rates of O_2_, CO_2_ CH_4_ and NH_4_^+^ in cocultures of *Methylacidiphilum* sp. RTK17.1 and *Galdieria* sp. RTK37.1 (Coculture) and their axenic controls. Negative values indicate consumption, while positive values indicate production. Coculture concentrations were estimated by considering each microorganism’s individual specific rate to be the same as in their axenic culture a) O_2_ specific production rates. b) CH_4_ specific production rates. For clarity, day 4 values for the coculture were omitted as their value drops due to methane depletion. c) CO_2_ specific production rates. d) NH_4_^+^ specific production rates. For clarity, day 4 values for the methanotroph and the microalgae were omitted since their value drops to 0 due to ammonium depletion. Cultures were grown in 1 L gastight bottles sideways on a shaking incubator at 45 °C, 110 rpm, and 60 μmol *m*^−2^ s^−1^ measured at the top of the bottle wall. The average value of duplicate experiments (*n* = 2) is shown with error bars representing one standard deviation.

Biomass normalised specific consumption and production rates (O_2_, CO_2_, CH_4_, NH_4_^+^) for the axenic cultures and the coculture were calculated (Fig. 4). *Methylacidiphilum* sp. RTK17.1 and *Galdieria* sp. RTK37.1 biomass concentrations in the coculture were estimated by performing a daily oxygen balance, under the assumption that the specific O_2_ uptake rates in the coculture were analogous to axenic cultures (Fig. 4a). Following complete CH_4_ consumption in the coculture, any further increase in OD_600_ was attributed solely to *Galdieria* sp. RTK37.1. Specific O_2_ production rates (Fig. 4a) were relatively stable for the coculture and axenic controls until the onset of nutrient limitation (O_2_ for the methanotroph, NH_4_^+^ for the microalgae, and CH_4_ for the coculture). Importantly, O_2_ specific uptake rates for *Methylacidiphilum* sp. RTK17.1 were ∼4-fold greater than microalgae specific production, which suggests that O_2_ production could become a limiting factor in higher density cocultures or increased methanotroph:microalgae ratios. When CH_4_ was depleted, the coculture’s O_2_ specific production approximated that of the axenic *Galdieria* control; presumably due to *Methylacidiphilum* sp. RTK17.1′s diminished activity in the absence of CH_4_. It is worth noting that this was also the case for CO_2_ (Fig. 4c) and NH_4_^+^ (Fig. 4d) specific consumption rates. Collectively, these data further suggested that *Galdieria* sp. RTK37.1 was not negatively affected by the presence of *Methylacidiphilum* sp. RTK17.1 in coculture.

Specific CH_4_ consumption rates indicated that enhanced CH_4_ consumption in the coculture was influenced both by an increase in *Methylacidiphilum* sp. RTK17.1 biomass and by an increase in the amount of CH_4_ consumed per mass unit of methanotroph (Fig. 4b). Presumably, the greater O_2_ availability in cocultures facilitated faster CH_4_ consumption rates. As expected, the specific rate of CH_4_ consumption, when normalised to coculture biomass, was slower than for the axenic methanotroph due to the majority of coculture biomass comprising microalgae. However, it was curious that this specific rate was constant. As there was almost no O_2_ in the headspace, it is likely methane oxidation was limited by O_2_ production, which is proportional to microalgae growth. With increasing *Galdieria* biomass, greater O_2_ availability for *Methylacidiphilum* RTK17.1 resulted in faster rates of CH_4_ oxidation for growth. Thus, CH_4_ oxidation in coculture incubation was reliant on microalgae growth. Such a phenomena has been observed previously with *M. alcaliphilum* biomass production rates being modulated by *Synechococcus* in an O_2_-limited chemostat coculture [21]. However, to test this hypothesis, a more accurate method to rapidly quantify the relative abundance of each microorganism would be required, as specific rates calculations are highly sensitive to inaccurate biomass measurements.

### 3.2 Supplemental O_2_ sparging mitigates benefits of oxygenic photosynthesis in chemostat coculture

To evaluate continuous coculture performance (and to generate sufficient biomass for amino acid characterization), chemostats were established for each axenic culture and the coculture (Table 1). The presence of *Galdieria* sp. RTK37.1 in the coculture increased the dissolved oxygen in the broth, and decreased volumetric O_2_ net consumption by 46 %, when compared to axenic *Methylacidiphilum* sp. RTK17.1. However, unlike the batch coculture, in chemostat the microalgae was unable to support the oxygen demand for the methanotroph; as the specific net O_2_ production was −0.54 mmol *h*^−1^ g_DW_^−1^. This observation was likely a consequence of the different *Galdieria:Methylacidiphilum* proportions in the chemostat; in the batch coculture experiments the *Galdieria:Methylacidiphilum* ratio was ∼ 3.6:1, while in the chemostat it was ∼1.3:1. Coculture chemostat operation, therefore required some external oxygen supplementation and contrasts prior studies where chemostat culture of *M. alcaliphilum* and *Synechococcus* did not require O_2_ supplementation because the photosynthetic O_2_ production was matched by O_2_ consumption [21]. This may have been a consequence of *Galdieria*’s slower O_2_ production rates, ∼120 µmol_oxygen_ mg_chlorophyll_^−1^ h^−1^ [52], compared to 710 µmol_oxygen_ mg_chlorophyll_^−1^ h^−1^ for *Synechococcus elongatus* PCC 7942 [53].

**Table 1:** Comparison of steady states values between axenic cultures and cocultures of *Methylacidiphilum* sp. RTK17.1 and *Galdieria* sp. RTK37.1 during chemostat cultivation.

Culture^a^	*Methylacidiphilum* sp. RTK17.1	Coculture	*Galdieria* sp. RTK37.1
**Chemostat conditions**			
Dilution rate (day^−1^)	0.167	0.167	0.278
Agitation (rpm)	250	250	400
**Steady state values**			
Concentration (g_DW_*L*^−1^)^c^	0.170 (± 0.006)	0.393 (± 0.014)	0.154 (± 0.002)
Biomass productivity (mg *L*^−1^ h^−1^)^c^	1.18 (± 0.04)	2.74 (± 0.10)	1.78 (± 0.02)
Dissolved oxygen (mg *L*^−1^)^b^	0.267 (± 0.005)	0.388 (± 0.009)	0.977 (± 0.009)
CH_4_ consumption^b^			
(mmol *L*^−1^ h^−1^)	0.242 (± 0.08)	0.211 (± 0.014)	–
(mmol *h*^−1^ g_DW_^−1^)	1.42 (± 0.05)	0.54 (± 0.04)	–
O_2_ net production^b^			
(mmol *L*^−1^ h^−1^)	−0.396 (± 0.032)	−0.214 (± 0.018)	0.149 (± 0.023)
(mmol *h*^−1^ g_DW_^−1^)	−2.33 (± 0.19)	−0.54 (± 0.05)	0.966 (± 0.152)
Biomass yield (g_DW_ mol_CH4_^−1^)^c^	4.89 (± 0.17)	13.0 (± 0.9)	–
CH_4_ removal ( %)^b^	40.5 (± 0.9)	34.3 (± 2.1)	–

a 1 L stirred tank reactors with 900 mL working volume, 45 °C and pH 2.5. Feed gas was supplied continuously at 16 mL min^−1^. Feed gas composition for *Methylacidiphilum* sp. RTK17.1 and the coculture were: 2.1 % O_2_, 1.1 % CH_4_, 74 % CO_2_, balance N_2_ (v/v). For *Galdieria* sp. RTK37.1 feed gas composition was 3.0 % O_2_, 64 % CO_2_, balance N_2_ (v/v). Systems were considered ‘steady state’ when there was < 5 % OD_600_ change over a five-day period. All chemostat cultures were N-replete.

b 12 day average after steady state was achieved. ^c^Biomass concentration of accumulated one day outflow broth, with *n* = 3 technical replicates. Values in brackets represent one standard deviation.

In the coculture chemostat, a steady-state biomass concentration of 0.393 g_DW_ *L*^−1^ and a biomass productivity of 2.74 mg *L*^−1^ h^−1^ were achieved, both values greater than their respective axenic counterparts. Biomass yields on methane in the *Methylacidiphilum* sp. RTK17.1 reactor were 4.89 ± 0.17 g_DW_ mol_CH4_^−1^; which is closer to the lower end of values typically reported for methanotrophs (2.4–24.48 g_DW_ mol_methane_^−1^[54]. In the coculture, the yield increased to 13.0 ± 0.9 g_DW_ mol_methane_^−1^ due to the inflationary contribution of *Galdieria* biomass. However, the biomass yield and specific CH_4_ consumption rates for *Methylacidiphilum* sp. RTK17.1 in chemostat were lesser than previously reported by Carere *et al*. [34]. Nevertheless, values were a consequence of the sub-optimal agitation rates used to accommodate the microalgae, which significantly impacted steady state biomass concentrations and methane removal. Decreasing agitation from 800 to 250 rpm resulted in OD_600_ declining from 0.92 to 0.29, and headspace CH_4_ removal from 87.2 % to 40.5 % in the *Methylacidiphilum* sp. RTK17.1 chemostat. For methanotroph reactors, increasing methane mass transfer is reported to have a major impact on biomass concentration, yields, and growth rates. For example, increasing the gas transfer coefficient in U-Loop reactors by a factor of 2.5 increased methanotroph concentration 3-fold [55]. Therefore, it is expected productivities reported in this study can be significantly improved via parameter optimisation of agitation, pressure and gas sparging rate.

The beneficial effect *Galdieria* sp. RTK37.1 exhibited on CH_4_ oxidation in batch was not observed in chemostat culture due to the supplemental delivery of O_2_ in coculture. As the *Methylacidiphilum* sp. RTK17.1 in coculture was not O_2_ limited, no benefit from the oxygenic activity of *Galdieria* sp. RTK37.1 was observed. The relative concentration of each microorganism may also have been an influencing factor, as photoautotroph:heterotroph inoculum ratio is a key factor contributing to mixed growth performance with microalgae [56]. This has also been observed for other types methanotroph cocultures, for example Jeong *et al*. [15] reported that the *Sphingopyxis* sp. NM1 stimulates growth and CH_4_ oxidation rates of *Methylocystis* sp. M6 when using a 1:9 M6:NM1 volumetric ratio; but not when the ratio was 9:1 or 1:1. It was found that *Sphingopyxis* enhanced transcriptional expression of genes involved in methane oxidation when it was more abundant than *Methylocystis*. A similar concentration dependant effect on methane oxidation was observed with mixed cultures of *Methylocystis* sp. M6 and *Hyphomicrobium* sp. NM3 [16].

### 3.3 Nutritional analysis reveals biomass is rich in essential amino acids

**Fig. 5:**
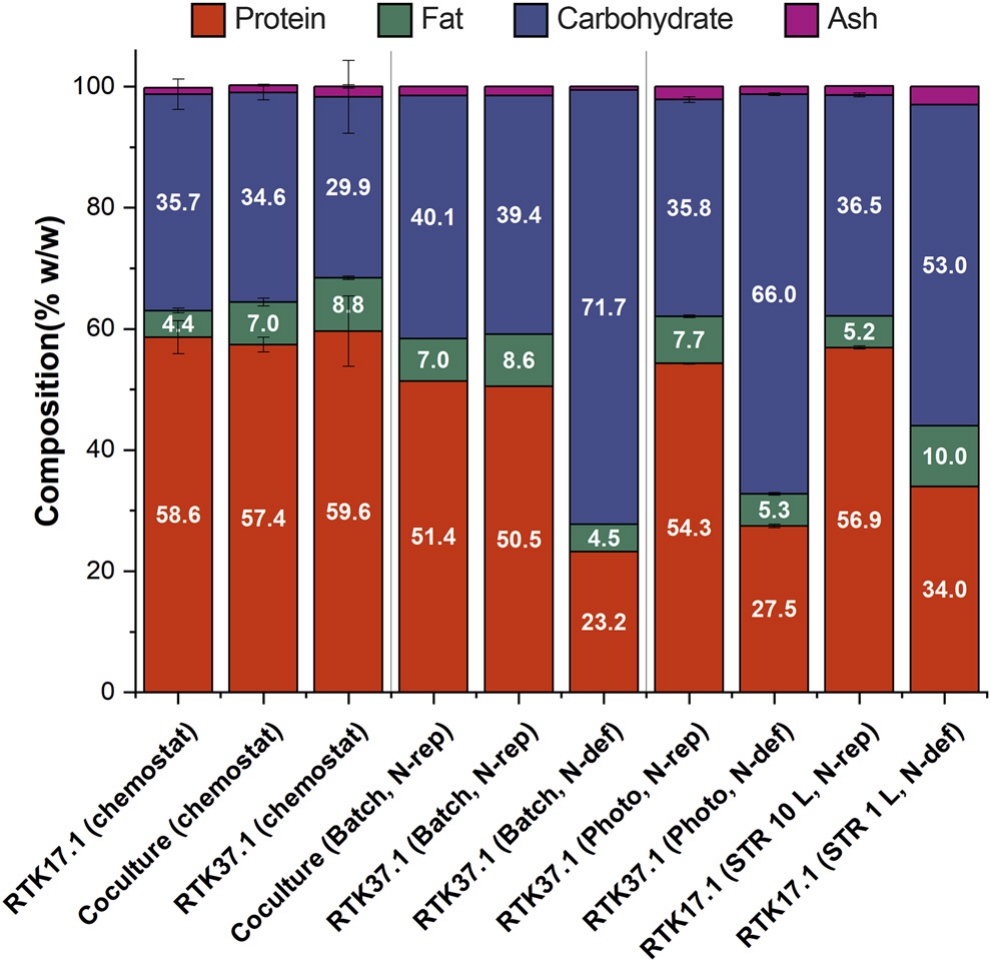
Macromolecular composition comparison between cultures and cocultures of *Methylacidiphilum* sp. RTK17.1 (RTK17.1) and *Galdieria* sp. RTK37.1 (RTK37.1). Chemostat refers to steady state bioreactors with conditions as referred in Table 1. ‘Batch’ refers to batch 1 L gastight bottles cultures. ‘Photo’ refers to 80 L photobioreactors, and ‘STR’ to stirred tank reactors, both with static liquid and continuous gas feed rate. N-rep refers to cultures harvested as soon as NH_4_^+^ was depleted, while N-def to cultures grown past NH_4_^+^depletion. For bottle cultures, duplicates samples were pooled together, and for the 1 L STR reactor only 1 sample was processed. For the remaining samples error bars represent 1 standard deviation for technical replicates (*n* = 3), except for RTK37.1 (chemostat) where *n* = 2.

To assess the suitability of *Methylacidiphilum* sp. RTK17.1 and *Galdieria* sp. RTK37.1 biomass as animal feeds, nutritional analysis of several cultures grown in batch (1-L gastight bottles), bioreactors with continuous gas flow (1-L STR, 10-L STR, and 80-L photobioreactor), or in chemostat (1-L STR reactors) was performed (Fig. 5). Non-nitrogen-limited cultures of *Methylacidiphilum* sp. RTK17.1, *Galdieria* sp. RTK37.1 and cocultures exhibited broadly similar macromolecular compositions, with protein content ranging from 50 % to 60 %, carbohydrates from 30 % to 40 %, fats from 4 % to 9 %, and ash ranging from 0.6 % to 3.0 % (all w/w). In comparison, the protein content of soybean meal is 44 % (w/w, crude protein) and fishmeal is 60 % (w/w, crude protein) [57]. The *Methylacidiphilum* sp. RTK17.1 chemostat would require 5.88 kg of methane to produce 1 kg of crude protein, while the coculture chemostat would require 2.15 kg of methane. In contrast, bacterial meal from *Methylococcus capsulatus* grown in a specially designed/optimised loop reactor requires 1.7 kg methane per kg of crude protein [58]. According to a techno-economic analysis, the projected production cost for *M. capsulatus* SCP ranges from 0.77 to 1.80 USD per kg of dry biomass, which translates to approximately 1.10 to 2.57 USD per kg of crude protein, assuming a protein content of 70 % [59].

The methanotroph cultures were slightly richer in carbohydrates and poorer in fats, while the coculture tended to have compositions closely resembling the axenic alga when grown in similar conditions. This is consistent with other *Galdieria* spp. cocultures. For example, Zhu *et al.* [44] reported *G. sulphuraria* grown mixotrophically in ultrahigh-NH_4_^+^ wastewater from industrial effluents, showed no significant difference in macromolecular contents when cultured in sterile or non-sterile media. However, the nutritional profile of the axenic *G. sulphuraria* (proteins 42.6 % to 50.8 %, lipids 21.9 % to 39.1 %, and carbohydrates 5.96 % to 6.02 % w/w) do contrast with our findings for *Galdieria* sp. RTK37.1. This can be explained by the mixotrophic mode of cultivation [60]. Heterotrophically grown cells of *G. sulphuraria* and *Galdieria partita* can be ∼30 % larger than autotrophically grown cells, and undergo important changes to internal cell structure [61,62]. Our findings also contrast with a reported consortium of *C. sorokiniana* and *M. capsulatus,* where the coculture displayed lower protein concentrations (28 % of the dry weight) than their respective axenic cultures (45 % and 53 % respectively), but higher fat contents (34 % for coculture, 30 % of the algae, and 22 % for the methanotroph) [20].

*Galdieria* sp. RTK37.1 and *Methylacidiphilum* sp. RTK17.1 grown for extended periods of ammonium depletion appreciably decreased their protein content and increased carbohydrate contents. This was consistent, as both microorganisms accumulate glycogen as a storage compound under nitrogen limitations [33,36]. Additionally, for microalgae nitrogen is necessary for continuous protein synthesis and indirectly for pigment formation [60], and in nitrogen-starved cells, free amino acid synthesis is slowed down [63].

With respect to amino acid composition, glutamic acid was the most concentrated amino acid in all cultures (Table S2). In chemostat, *Methylacidiphilum* sp. RTK17.1 was significantly richer than *Galdieria* sp. RTK37.1 in histidine, methionine, and phenylalanine (*p*-values < 0.05). Conversely, the microalgae displayed greater concentrations of threonine, cysteine, tyrosine, and serine (*p*-values < 0.05). For the coculture, no amino acid concentration significantly varied from axenic cultures (*p*-value > 0.05), aside from tyrosine and cysteine. The chemostat coculture had an essential amino acid concentration of 20.84 ± 0.90 g/100 g_DW_ which is greater than the values reported for *Methyloparacoccus murrelli* LMG 27,482 in coculture with *Cupriavidus necator* LMG 1201 (14.5 g/100 g_DW_, [55].

**Fig. 6:**
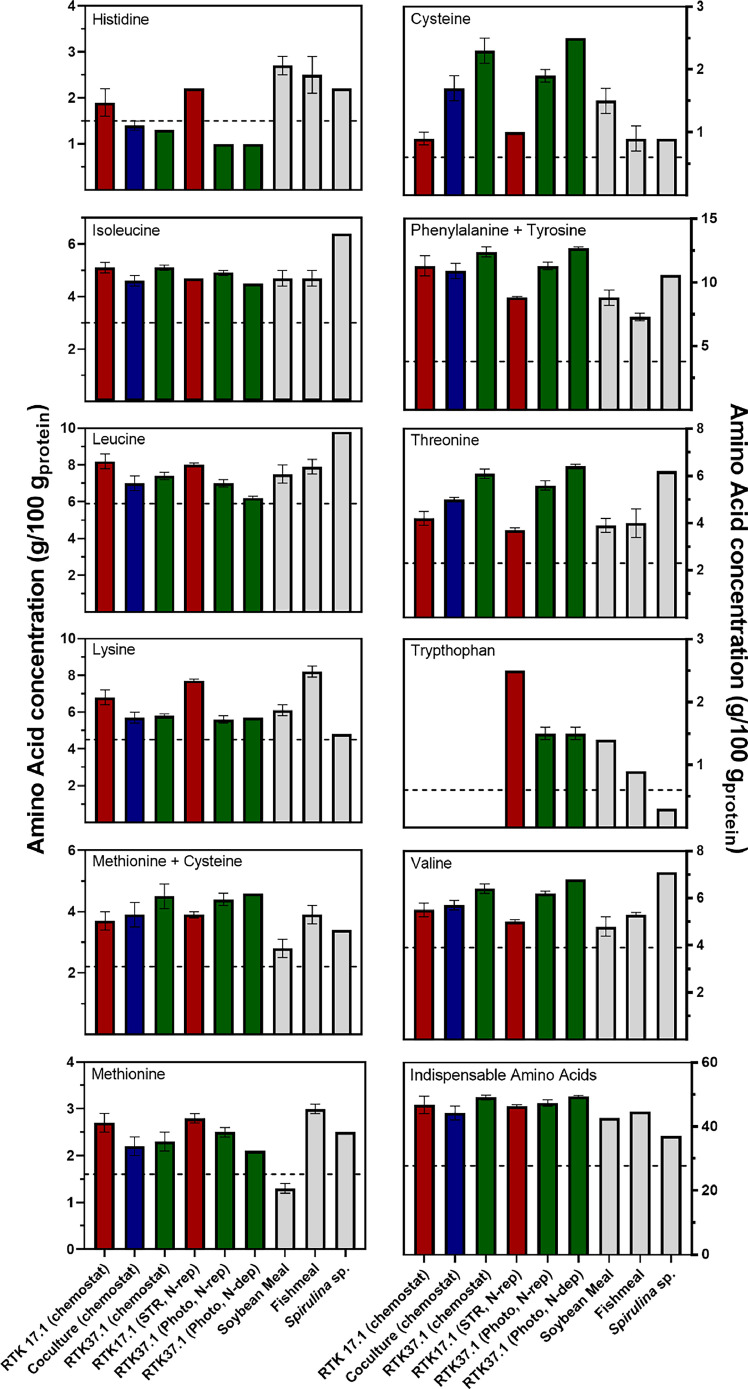
Indispensable amino acid composition comparison between cultures and cocultures of *Methylacidiphilum* sp. RTK17.1 and *Galdieria* sp. RTK37.1, and reference proteins. Chemostat refers to steady state bioreactors with conditions as referred in Table 1. ‘Photo’ refers to 80 L photobioreactors, and ‘STR’ to stirred tank reactors, both with static liquid and continuous gas feed rate. ‘N-rep’ refers to cultures harvested as soon as NH_4_^+^ was depleted, while ‘N-def’ to cultures grown past NH_4_^+^depletion. Average values are shown with error bars representing one standard deviation (*n* = 3 for all biomass samples, except for RTK37.1 (chemostat) where *n* = 2). Bars are colour coded red for *Methylacidiphilum* sp. RTK17.1, green for *Galdieria* sp. RTK37.1, blue for cocultures, and grey for reference proteins. The horizontal dashed line represents the FAO/WHO/UNU reference protein. Reference proteins values for soybean meal and fishmeal were taken from Overland et al. (2010), and *Spirulina* from Becker (2007).

The FAO/WHO/UNU standard describes the required daily intake of specific amino acids by humans and is often used to evaluate whether a protein source can provide an adult all the required indispensable amino acids [64]. Soybean meal and fishmeal are common proteins used as animal feed [55]. In Fig. 6, the indispensable amino acid profiles of produced biomass, along with soybean meal, fishmeal and the FAO/WHO/UNU standard are provided. While protein and carbohydrate content for each microorganism differed depending on growth condition (Fig. 5), amino acid profiles remained largely unchanged. It is often the case for both microalgae and methanotroph biomass that growth conditions largely affect macromolecular composition, but not the amino acid profile [58,65,66]. For *Methylacidiphilum* sp. RTK17.1, phenylalanine and tyrosine (11.3 g/g_protein_ in chemostat vs 8.8 g/g_protein_ in a 10 L STR) exhibited the greatest difference. For *Galdieria* sp. RTK37.1*,* leucine concentration went from 7.0 g/g_protein_ in chemostat to 6.2 g/g_protein_ in the ammonium depleted photobioreactor, and there was < 0.5 % difference in the concentrations of histidine, cysteine, and methionine between reactor systems. The protein content of *Methylacidiphilum* sp. RTK17.1, *Galdieria* sp. RTK37.1, and the cocultures were comparable in quality to both fishmeal and soybean meal (Fig. 6). The methanotroph protein meets all the FAO/WHO/UNU reference protein concentrations, it also surpasses all indispensable amino acid concentrations of soybean meal and fishmeal, with the exceptions of cysteine in soybean meal, and histidine in both reference proteins. The microalgae and coculture proteins were marginally deficient in histidine when compared to the FAO/WHO/UNU reference protein, displayed lesser lysine and methionine concentrations than fishmeal but otherwise were comparable (or superior) regarding indispensable amino acid content.

Lysine is the main limiting amino acid for animal species fed grains as a main energy source [67,68]; with most plant-based proteins displaying relatively poor lysine contents (3.6 ± 0.6 %) and methionine (3.6 ± 0.6 %) [69]. This makes the proteins from *Methylacidiphilum* sp. RTK17.1 and *Galdieria* sp. RTK37.1 attractive as replacement feedstocks, as they had greater quantities of methionine and cysteine (compared to soybean meal and fishmeal), and the methanotroph lysine content was comparable to fishmeal and higher than soybean meal. In comparison, lysine was deficient in bacterial protein meals produced by a consortium of *M. capsulatus* (Bath), *Alcaligenes acidovorans, Bacillus brevis*, and *Bacillus firmus* grown on natural gas [68]. Lysine was also limiting in most cocultures of methanotrophs with hydrogen-oxidizing bacteria [55]. Methionine was deficient in most analysed microalgae [70], and in commercial *Spirulina* [71]. A community of methanotrophs dominated by *Methylomonas* and *Methylocystis* spp. lacked sufficient concentrations of both lysine and methionine [72,73]. Kerckhof *et al.* [55] calculated that the amount of microbial biomass needed to meet the individual amino acid requirements for an adult human (62 kg) ranged from 139 g, for a coculture of *Methyloparacoccus murreli* with *Cupriavidus necator* (lysine being the limiting amino acid) to 1982 g for an axenic culture of *Hydrogenophaga electrium* (limited by isoleucine). For our chemostat cultures, a similar calculation revealed that histidine was the limiting amino acid for all cultures, and the biomass required to satisfy amino acid requirements in humans, would be 568 g for *Methylacidiphilum* sp. RTK17.1, 804.8 g *Galdieria* sp. RTK37.1, and 753.7 g for the coculture.

### 3.4 Limitations

This study provides insight into the dynamics of methanotroph-photoautotroph coculture systems; however, several limitations must be acknowledged. Biomass concentrations of each microorganism in coculture were indirectly estimated via daily oxygen balance, assuming that specific oxygen uptake and production rates remained constant throughout cultivation. This approximation could become less reliable under nutrient limited conditions, where metabolic shifts may alter O₂ dynamics. Additionally, this could also be the case if there are positive or negative interactions between microorganisms [74].

Studies examining methanotroph–photoautotroph coculture dynamics frequently report aggregate biomass concentrations without distinguishing the individual contributions of each microorganism [20,21]. While flow cytometry is occasionally employed to provide direct cell counts and relative abundance estimates [21,75], its use is not routine. Some investigations apply multi-method approaches—such as combining microscopy, qPCR, and spectroscopy—to quantify each population within the coculture [55]. However, these approaches tend to be labor-intensive, lack real-time capability, and often require access to specialized instrumentation. Therefore, there is a need to develop rapid, low-cost laboratory methods that exploit inherent physical properties of microorganisms (e.g., cell size, morphology, density, and autofluorescence) to estimate their relative abundance within cocultures more efficiently.

Furthermore, the findings underscore oxygen availability as a critical limiting factor in coculture performance, particularly as broth concentrations and methanotroph abundance increase. In dense cultures, oxygen demand could outpace microalgal oxygen production, limiting growth [20,44,76]. This presents a key challenge for scale-up, where maintaining adequate O₂ levels is more complex due to limitations in gas–liquid mass transfer, mixing efficiency, and light penetration. Although gas transfer rates can be improved by increasing agitation or pressure [72,77], such modifications may also increase energy costs or negatively affect shear-sensitive *Galdieria* cells.

Additionally, the coculture's performance metrics (e.g., biomass yields, CH₄ conversion efficiency) remain lower than those reported for other methanotroph–photoautotroph systems. Improving these metrics may require exploring alternate reactor configurations, such as photobioreactors with enhanced light distribution, or sequential cultivation strategies where each microorganism is grown under optimal, separate conditions [55,78,79].

## 4 Conclusions

This study demonstrated a ‘proof-of-concept’ that the thermoacidophiles, *Methylacidiphilum* sp. RTK17.1 and *Galdieria* sp. RTK37.1, could be stably grown in coculture via batch and continuous operation. During batch growth, the methanotroph benefited from the interaction when in a microaerobic environment, as seen by the increased growth and methane oxidation rates. At the methanotroph:microalgae proportions investigated, no evidence of *Galdieria* sp. RTK37.1 being negatively affected by the presence of *Methylacidiphilum* sp. RTK17.1 was detected. However, increasing the relative concentration of methanotroph could introduce a shading effect, which could limit photosynthesis, and impact coculture growth. This could also be the case for greater concentration broths in general, as shading effects and greater oxygen demands could further limit O_2_ availability. During continuous cultivation, the beneficial effect *Galdieria* RTK37.1 exhibited on CH_4_ oxidation in batch was not observed due to the supplemental delivery of O_2_ in coculture. Oxygen played an important role in *Methylacidiphilum*-*Galdieria* interactions, but to study it further, a method to rapidly quantify the abundance of each microorganism, that does not rely on gas uptake rates, is required. According to amino acid profiles, *Methylacidiphilum* sp. RTK17.1 and *Galdieria* sp. RTK37.1 biomass each exhibited a similar nutritional quality to soybean meal and fishmeal and could potentially be used as a high protein content feed for animals.

## Data Availability Statement

All data may be made available by request.

## Declaration of Competing Interest

The authors declare the following financial interests/personal relationships which may be considered as potential competing interests:

A patent application (PCT/IB2024/054,938) has been submitted and is currently in consideration. All authors declare that they have no known competing financial interests or personal relationships that could have appeared to influence the work reported in this paper.

## Appendix Supplementary materials

Image, application 1
