# Supplementary material for: Simultaneous co-cultivation of the thermoacidophilic methanotroph, Methylacidiphilum sp. RTK17.1, and the microalga, Galdieria sp. RTK37.1, for single cell protein production
Source: Eng Microbiol. 2025 Aug 6;5(4):100229. doi: 10.1016/j.engmic.2025.100229 (PMC12967838; doi:10.1016/j.engmic.2025.100229)
Supplement: Supplementary file 1 [file mmc1.docx]

**Table S1.** Initial CO_2_, CH_4_, and O_2_ headspace gas concentrations for the batch coculture experiments.

| **Culture** | **Headspace concentration^a^ (% v/v)** | | |
| --- | --- | --- | --- |
|  | **O_2_** | **CH_4_** | **CO_2_** |
| ***1. Methylacidiphilum* sp. RTK17.1 (axenic)** | **13.44 ± 0.02** | **11.96** ± **0.33** | **5.12 ± 0.18** |
| ***2.* Coculture** | **1.38** ± **0.19** | **12.37 ± 1.28** | **64.05 ± 4.05** |
| ***3. Galdieria* sp. RTK37.1 (axenic)** | **1.41** ± **0.01** | **0** | **77.90 ± 2.36** |

**^a^ Balance N_2_ for all cultures.**

**Table S2.** Amino acid distribution for cultures and cocultures of *Methylacidiphilum* sp. RTK17.1 and *Galdieria* sp. RTK37.1 in a chemostat, or batch cultures (stirred tank reactor (STR) or photobioreactor (photo)). In batch cultures, media were either nitrogen replete (N-rep) or nitrogen deficient (N-def)

| Amino Acid  (g/100 g_DW_) | RTK17.1^a,c^  (chemostat) | Coculture^c^  (chemostat) | RTK37.1^b,d^  (chemostat) | RTK17.1^a,e^  (STR, N-rep) | RTK37.1^b,f^  (photo, N-rep) | RTK37.1^b,g^  (photo, N-def) |
| --- | --- | --- | --- | --- | --- | --- |
| **Essential amino acids (****g/100 g_DW_)^h^** | | | | | | |
| Histidine | 1.12 (± 0.10) | 0.82 (± 0.07) | 0.77 (± 0.09) | 1.25 (± 0.03) | 0.54 (± 0.01) | 0.29 (± 0.01) |
| Isoleucine | 2.99 (± 0.01) | 2.64 (± 0.10) | 3.04 (± 0.37) | 2.68 (± 0.04) | 2.64 (± 0.03) | 1.24 (± 0.01) |
| Leucine | 4.82 (± 0.00) | 3.99 (± 0.19) | 4.41 (± 0.55) | 4.54 (± 0.07) | 3.80 (± 0.07) | 1.70 (± 0.02) |
| Lysine | 4.01 (± 0.17) | 3.29 (± 0.22) | 3.48 (± 0.39) | 4.36 (± 0.09) | 3.05 (± 0.08) | 1.57 (± 0.01) |
| Methionine | 1.59 (± 0.02) | 1.27 (± 0.08) | 1.35 (± 0.00) | 1.62 (± 0.03) | 1.33 (± 0.05) | 0.57 (± 0.01) |
| Phenylalanine | 3.66 (± 0.12) | 2.68 (± 0.24) | 2.71 (± 0.37) | 2.67 (± 0.02) | 2.25 (± 0.06) | 1.12 (± 0.02) |
| Threonine | 2.47 (± 0.05) | 2.88 (± 0.07) | 3.63 (± 0.45) | 2.12 (± 0.04) | 3.02 (± 0.08) | 1.76 (± 0.02) |
| Tryptophan | NR^i^ | NR^i^ | NR^i^ | 1.40 (± 0.03) | 0.82 (± 0.04) | 0.42 (± 0.02) |
| Valine | 3.20 (± 0.01) | 3.25 (± 0.09) | 3.84 (± 0.48) | 2.83 (± 0.04) | 3.34 (± 0.03) | 1.86 (± 0.01) |
| Essential AA | 23.87 (± 0.28) | 20.84 (± 0.90) | 23.23 (± 2.69) | 23.47 (± 0.32) | 20.79 (± 0.40) | 10.52 (± 0.05) |
| **Non essential amino acids (g/100 g_DW_)^h^** | | | | | | |
| Cysteine | 0.54 (± 0.04) | 0.99 (± 0.13) | 1.34 (± 0.01) | 0.59 (± 0.01) | 1.06 (± 0.05) | 0.69 (± 0.01) |
| Tyrosine | 2.96 (± 0.03) | 3.60 (± 0.06) | 4.69 (± 0.62) | 2.33 (± 0.04) | 3.87 (± 0.06) | 2.37 (± 0.02) |
| Glycine | 3.14 (± 0.13) | 2.31 (± 0.12) | 2.36 (± 0.33) | 2.67 (± 0.14) | 2.09 (± 0.01) | 0.96 (± 0.03) |
| Glutamic acid | 6.69 (± 0.58) | 7.04 (± 0.53) | 8.08 (± 0.93) | 7.84 (± 0.19) | 6.68 (± 0.65) | 3.92 (± 0.05) |
| Serine | 2.49 (± 0.22) | 3.12 (± 0.13) | 4.04 (± 0.54) | 2.13 (± 0.06) | 3.29 (± 0.23) | 1.76 (± 0.03) |
| Aspartic Acid | 4.45 (± 0.19) | 4.15 (± 0.24) | 4.95 (± 0.59) | 4.47 (± 0.04) | 4.14 (± 0.19) | 1.78 (± 0.05) |
| Arginine | 2.77 (± 0.07) | 2.75 (± 0.10) | 3.40 (± 0.40) | 2.80 (± 0.05) | 3.11 (± 0.05) | 1.26 (± 0.02) |
| Taurine | 0.05 (± 0.00) | 0.03 (± 0.00) | 0.07 (± 0.00) | NR^i^ | 0.13 (± 0.01) | 0.05 (± 0.01) |
| Proline | 2.52 (± 0.04) | 2.66 (± 0.12) | 3.13 (± 0.35) | 2.21 (± 0.03) | 2.36 (± 0.05) | 1.45 (± 0.01) |
| Alanine | 3.22 (± 0.04) | 2.92 (± 0.12) | 3.42 (± 0.43) | 2.95 (± 0.04) | 2.83 (± 0.08) | 1.26 (± 0.01) |

^a^ RTK17.1 stands for *Methylacidiphilum* sp. RTK17.1. ^b^ RTK37.1 stands for *Galdieria* sp. RTK37.1. ^c^1 L stirred tank reactor, dilution rate 0.167 day^-1^, and 250 rpm agitation rate. ^d^1 L stirred tank reactor, dilution rate 0.278 day^-1^, and 400 rpm agitation rate. ^e^10 L stirred tank reactor static liquid and continuous gas feed rate, with intermittent NH_4_^+^ additions. ^f^40 L tubular photobioreactor, static liquid and continuous gas feed rate, cultivated until NH_4_^+^ depletion. ^g^40 L photobioreactor, static liquid and continuous gas feed rate, cultivated 7 days past NH_4_^+^ depletion. ^h^Values in brackets are standard deviations with n = 3 technical replicates for all biomass samples, except for RTK37.1 (chemostat) with n = 2. ^I^ NR, no result, amino acid not measured.
